# Probable late lyme disease: a variant manifestation of untreated *Borrelia burgdorferi* infection

**DOI:** 10.1186/1471-2334-12-173

**Published:** 2012-08-01

**Authors:** John N Aucott, Ari Seifter, Alison W Rebman

**Affiliations:** 1 Department of Medicine, Division of General Internal Medicine, Johns Hopkins University School of Medicine, Baltimore, MD, USA; 2 The Lyme Disease Research Foundation of Maryland, Lutherville, MD, USA

## Abstract

**Background:**

Lyme disease, a bacterial infection with the tick-borne spirochete *Borrelia burgdorferi*, can cause early and late manifestations. The category of probable Lyme disease was recently added to the CDC surveillance case definition to describe patients with serologic evidence of exposure and physician-diagnosed disease in the absence of objective signs. We present a retrospective case series of 13 untreated patients with persistent symptoms of greater than 12 weeks duration who meet these criteria and suggest a label of ‘probable late Lyme disease’ for this presentation.

**Methods:**

The sample for this analysis draws from a retrospective chart review of consecutive, adult patients presenting between August 2002 and August 2007 to the author (JA), an infectious disease specialist. Patients were included in the analysis if their current illness had lasted greater than or equal to 12 weeks duration at the time of evaluation.

**Results:**

Probable late Lyme patients with positive IgG serology but no history of previous physician-documented Lyme disease or appropriate Lyme treatment were found to represent 6% of our heterogeneous sample presenting with ≥ 12 weeks of symptom duration. Patients experienced a range of symptoms including fatigue, widespread pain, and cognitive complaints. Approximately one-third of this subset reported a patient-observed rash at illness onset, with a similar proportion having been exposed to non-recommended antibiotics or glucocorticosteroid treatment for their initial disease. A clinically significant response to antibiotics treatment was noted in the majority of patients with probable late Lyme disease, although post-treatment symptom recurrence was common.

**Conclusions:**

We suggest that patients with probable late Lyme disease share features with both confirmed late Lyme disease and post-treatment Lyme disease syndrome. Physicians should consider the recent inclusion of probable Lyme disease in the CDC Lyme disease surveillance criteria when evaluating patients, especially in patients with a history suggestive of misdiagnosed or inadequately treated early Lyme disease. Further studies are warranted to delineate later manifestations of Lyme disease and to quantify treatment benefit in this population.

## Background

Lyme disease, caused by infection with the tick-borne spirochete *Borrelia burgdorferi*, is the most common vector-borne disease in North America. In highly endemic regions of the United States, the annual incidence of infection may be as high as 1–3% with a cumulative prevalence as high as 7–15% [1,2]. In the majority of cases, the diagnosis of confirmed early Lyme disease is based on identification of the hallmark erythema migrans (EM) rash, which may occur in isolation or in conjunction with viral-like symptoms such as fever, malaise, fatigue, and generalized achiness [3].

However, in up to 16% of early Lyme cases, patients do not present with a rash and the primary symptoms of their acute illness are viral-like [4]. Historically, surveillance criteria requiring a high degree of specificity have excluded patients with only viral-like or subjective patient-reported symptoms in case definitions for Lyme disease [5]. In 2008, however, the Centers for Disease Control (CDC) surveillance criteria for Lyme disease were modified to include patients with solely subjective symptoms and a positive confirmatory serology as probable Lyme disease [6]. Based on the revised surveillance case definition, more than 30,000 new confirmed or probable cases of Lyme disease were reported in 2009 [7], though studies have shown that the actual number of cases may exceed reported cases by a factor of 6 to 12 in endemic areas [8,9].

If left untreated, Lyme disease may progress to later stages involving the musculoskeletal, neurologic, or cardiovascular systems. The diagnosis of these late stages of Lyme disease is based on clinical diagnosis with serologic confirmation using CDC surveillance criteria [10]. The CDC case definition for confirmed late Lyme disease relies on signs of specific organ damage such as inflammatory arthritis with synovitis and joint effusion, or objective neurologic disease, all confirmed by a positive IgG immunoblot (western blot) for antibodies to *B. burgdorferi*[6]. However, an initial longitudinal observation of untreated Lyme disease patients suggested that a significant number (18%) of late Lyme cases may only exhibit symptoms such as fatigue, arthralgias or myalgias, without development of classic physical signs of late Lyme arthritis or neurologic disease [11]. The patient phenotype of IgG seropositivity and musculoskeletal pain, fatigue, and/or cognitive dysfunction without signs of organ inflammation or dysfunction corresponds with a late manifestation of the current surveillance category of probable Lyme disease. When present, this phenotype can be termed “probable late Lyme disease”.

The specific presentation of probable late Lyme disease, including untreated patients with a history of subjective symptoms and a positive IgG immunoblot, differs in two important ways from those meeting criteria for post-treatment Lyme disease syndrome (PTLDS), a disease category recently added to the Infectious Disease Society of America (IDSA) guidelines [10]. First, patients with probable late Lyme have no history of a prior physician diagnosis of objective findings consistent with early or late Lyme disease, a requirement for patients with PTLDS. Second, patients with probable late Lyme have not been previously treated with an antibiotic regimen recommended for Lyme disease, also a requirement for PTLDS. Since patients with probable late Lyme disease have serologic evidence of remote exposure to *B. burgdorferi*, they represent a distinct subset among patients with other chronic presentations that are often categorized as “medically unexplained” symptoms or syndromes such as chronic fatigue syndrome or fibromyalgia.

The existence of probable late Lyme disease, manifesting only as subjective symptoms with a concurrent positive IgG immunoblot serology, has remained controversial. This patient presentation was described in a recent review categorizing the spectrum of patients labelled as having “chronic Lyme disease” [12]. The authors suggest that IgG seropositive patients with symptoms but no signs of illness have at most “equivocal evidence for infection with *B. burgdorferi* and that any benefit from treatment would be unlikely” [12]. Patients with probable late Lyme disease share clinical phenotypes which overlap with patients who have *PTLDS*, fibromyalgia, chronic fatigue syndrome, and those whose symptoms remain medically unexplained after extensive medical evaluation. Thus, many have argued against serologic screening for Lyme disease among patients whose symptoms of fatigue, widespread pain, and subjective cognitive dysfunction exist in the absence of physical findings or laboratory abnormalities [2,12].

Patients with probable late Lyme disease, or those untreated patients with a positive IgG serologic test for Lyme disease and otherwise unexplained symptoms, represent an interesting subset of patients who have not been clinically characterized in the modern Lyme disease literature. This article offers a description of a sample of such patients, within the context of the wider spectrum of late and chronic Lyme patients seen for evaluation in community-based clinical practice.

## Methods

### Patients and setting

The sample for this analysis draws from a chart review of consecutive, adult patients age 18 and above referred for evaluation of possible Lyme disease between August 2002 and August 2007 to the author (JA), an infectious disease trained internist. The practice is located in a Lyme-endemic suburban community of a medium sized Mid-Atlantic city. Patients had either been self or physician referred for consultation and in some cases had already been treated with antibiotics for Lyme disease prior to their evaluation.

At the time of medical evaluation, a complete history was taken for all patients and a physical exam was performed. All objective physical exam findings and subjective symptoms were documented and any relevant medical records were reviewed. All records of acute and convalescent ELISA and immunoblot serologies from commercial laboratories utilizing CDC interpretive criteria were documented [13]. Immunoblot results were included even if a simultaneous ELISA was not documented in the records. Extensive evaluation for alternative diagnoses was initiated if the medical history, physical exam, or laboratory findings were suggestive of such. Patients self-reported prior diagnoses of their current illness as well as any antibiotic or glucocorticosteroid use following disease onset. Charts were later abstracted and relevant clinical information was entered into a database. The database was used to identify patients with probable late Lyme disease; these charts were individually reviewed to obtain further information on the patients presenting symptoms and their subsequent treatment response.

### Definition of cases

Patients were included in the analysis if their current illness had lasted greater than or equal to 12 weeks duration at the time of evaluation. This subset was then characterized as meeting criteria for one of five disease categories, corresponding to those identified by Feder et al. [12]. Final group determination was reviewed by a clinician (JA) to ensure accuracy.

 a) *Confirmed late Lyme disease*. CDC guidelines for diagnosis of confirmed late Lyme arthritis, carditis, or neurologic disease, including the presence of objective signs involving the joints, the heart, or the central or peripheral nervous system, were stringently applied [6].

 b) *Other, non-Lyme diagnoses*. Patients diagnosed with an alternative, non-Lyme diagnosis which explained their presenting symptoms were identified in this group.

 c) *Probable late Lyme disease*. Patients in this group were defined by a positive IgG serologic test and a history of self reported subjective symptoms including some combination of fatigue, non-inflammatory musculoskeletal pain, and cognitive symptoms. Patients with a history of either a physician-documented EM rash or other objective finding suggestive of Lyme disease were excluded. Patients were also excluded from this category if evidence was found that they had been treated with a recommended antibiotic regimen for early Lyme disease. However, patients with non-recommended antibiotic, duration of antibiotic therapy less than 7 days, and glucocorticoid exposure for presumed alternate diagnoses were included.

 d) PTLDS. Patients meeting all criteria for the IDSA proposed definition of PTLDS were included in this group [10]. Patients meeting a less stringent definition with a history of physician-documented Lyme disease and current fatigue, musculoskeletal pain or neurocognitive dysfunction but failing to meet supplementary inclusion or exclusion criteria relating to the timing of symptom onset or the presence of minor depression were also included, but are identified and presented separately.

 e) *No specific diagnosis*. The remaining patients who did not meet criteria for any of the above categories were characterized as a final group with medically unexplained symptoms and with no specific diagnosis.

### Clinical characteristics of probable late lyme disease

Demographic and clinical characteristics of patients meeting criteria for probable late Lyme were then determined. Illness duration was defined from initial patient-reported date of onset of subjective symptoms coinciding with the present illness. Charts were also reviewed for symptoms reported; symptoms were considered present as part of the current illness if mentioned in the medical record after date of illness onset. While group criteria excluded those with a history of physician-documented EM, patients with self-observed rashes either not observed by a physician, or thought not to be EM, were included. Antibiotics considered non-recommended or of insufficient duration for treatment of Lyme disease included all members of the quinolone class of antibiotics, first generation cephalosporins, and five-day courses of azithromycin [10].

Tests for group differences were performed using chi-square or Kruskal-Wallis tests, as appropriate. Results with a two-sided p < 0.05 were considered statistically significant.

This chart review was approved by the Institutional Review Board of the Johns Hopkins University School of Medicine.

## Results

### Disease classification

**Figure 1:**
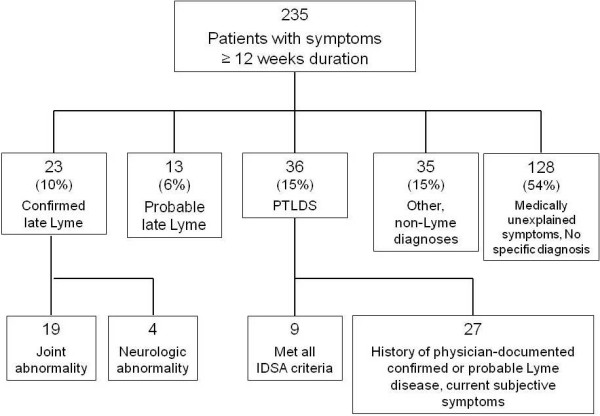
Categorization of patients presenting for evaluation of Lyme disease with symptoms ≥ 12 weeks duration (n = 235).

A total of 235 patients with symptoms greater than 12 weeks were seen for evaluation (Figure 1). Of these, 35 (15%) had alternative, non-Lyme diagnoses which explained their presenting signs and symptoms. An additional 128 (54%) could not be assigned a specific diagnosis or category using the current CDC surveillance criteria for Lyme disease and had medically unexplained symptoms.

Among the remaining patients, 36 (15%) had a history of prior, physician-documented and treated early Lyme disease with persistent or recurrent symptoms. Nine of these (25%) met the proposed IDSA case definition of PTLDS, while the remaining 27 (75%) had persistent symptoms after treatment of Lyme disease but failed to meet supplementary inclusion or exclusion criteria in the IDSA case definition. The most common reasons for exclusion were the presence of an exclusionary comorbid condition which preceded illness onset (12/27, 44%), a symptom duration of less than 6 months (7/27, 26%), and a greater than six month duration between exposure and subjective illness onset (7/27, 26%, mean time to onset of illness 3.5 years, range 2.1-5.5 years).

Twenty-three patients (10%) met criteria for confirmed late Lyme disease, with the majority presenting with objective oligoarthritis, primarily of the knee, rather than neurologic abnormalities. The remaining 13 patients (6%) met the definition of probable late Lyme disease with patient-reported symptoms and positive IgG immunoblot, but without a history of previously physician-documented Lyme disease or prior appropriate treatment. Probable late Lyme accounted for 12% of those with medically explained symptoms and a specific Lyme or non-Lyme related diagnosis.

### Probable late lyme disease

**Figure 2:**
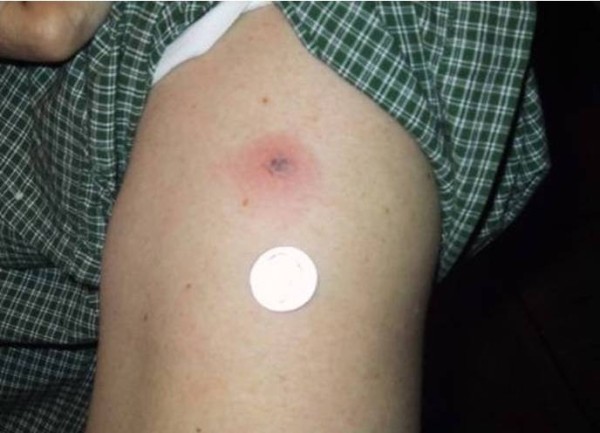
**Untreated skin lesion in patient number 1. Because of the small size, lack of enlargement and lack of symptoms the lesion was not felt to be consistent with erythema migrans.** The patient noted onset of symptoms 10 weeks later with olecranon bursitis.

Table 1 presents demographic and clinical characteristics of the 13 patients with probable late Lyme disease in comparison to patients with other Lyme disease and non-Lyme disease related diagnoses. The median age at illness onset was 61 in patients with probable late Lyme disease. The majority of the patients in the overall sample, with exception of those with confirmed late Lyme arthritis, presented with some combination of fatigue, generalized musculoskeletal pain, and cognitive complaints. By definition, patients with probable late Lyme disease had no history of physician documented or treated EM rash and no objective clinical signs associated with late Lyme disease. However, 38% recalled a rash consistent with EM at illness onset not documented or diagnosed by a physician. In some cases the rash was felt by the patient or physician to be atypical for Lyme disease because of the lack of the classic “bull’s eye” appearance or because the rash did not meet the minimum 5 cm size in the CDC surveillance criteria (Figure 2, patient #1 Table 2). This proportion of prior patient-documented rash in the absence of physician diagnosed erythema migrans was higher in the probable late Lyme disease group when compared to the other four disease groups (4% late Lyme, 3% PTLDS, 14% other diagnoses, 26% no specific diagnosis, p = 0.002).

**Table 1:** Demographic and clinical characteristics of patients included in this retrospective chart review*

**Characteristics**	**Confirmed Late (n=23)**	**Probable Late (n=13)**	**PLS (n=36)**	**Other diagnosis (n=35)**	**No specific diagnosis (n=128)**
Demographics					
Age at illness onset	49.1 (44.9-65.3)	56.6 (50-67)	46.6 (35.5-52.5)	55.4 (36.5-61.6)	42.2 (32.1-53.0)
Male Sex	14/23 (61%)	6/13 (46%)	19/36 (53%)	14/35 (40%)	43/128 (34%)
Illness duration (weeks)	52 (9-130)	63 (52-152)	127 (30-270)	52 (25-216)	78 (40-204)
Symptoms					
Widespread musculoskeletal pain	2/23 (9%)	11/13 (85%)	36/36 (100%)	17/35 (49%)	112/128 (88%)
Fatigue	0/23 (0%)	8/13 (62%)	32/36 (89%)	15/35 (43%)	96/128 (75%)
Cognitive complaints	0/23 (0%)	6/13 (46%)	19/36 (53%)	8/35 (23%)	67/128 (52%)
Signs					
Patient reported rash in absence of physician diagnosis of EM	1/23 (4%)	5/13 (38%)	1/36 (3%)	5/35 (14%)	33/128 (26%)
Physician documented EM	1/23 (4%)	0/13 (0%)	26/36 (72%)	4/35 (11%)	1/128 (1%)
Other physician documented objective findings	21/23 (91%)	0/13 (0%)	9/36 (25%)	2/35 (6%)	11/128 (9%)
Serology					
% ELISA positive ( >.9)	17/18 (94%)	11/11 (100%)	18/28 (64%)	6/23 (26%)	6/104 (6%)
Median ELISA among those (+)	3.5	2.0	1.8	1.75	1.4
% IgM immunoblot (+)	14/22 (64%)	2/11 (18%)	14/31 (45%)	4/28 (14%)	32/109 (29%)
Treatment					
Non-recommended antibiotic	8/23 (35%)	4/13 (31%)	14/36 (39%)	6/35 (17%)	43/128 (34%)
Steroid	3/23 (13%)	2/13 (15%)	9/36 (25%)	5/35 (14%)	19/128 (15%)

*no. (%) for dichotomous variables and median (IQR) for continuous variables.

**Table 2:** Specific symptoms and responses to therapy of the 13 patients with probable late Lyme disease

**Patient**	**Exposure History**	**Presenting Symptom/signs**	**Duration Symptoms**	**Treatment**	**Outcome**
1	Non-diagnostic skin lesion not treated. (See figure 2)	Arthralgias, neck stiffness, fatigue; Olecranon bursitis	12 months	Doxycycline 4 weeks	All symptoms resolved. No Relapse
2	Unknown	Arthralgias: hip; Headache	5 years	Doxycycline 7 weeks	80% Improved. No relapse
3	Tick bite with cellulitis Rx with cephalexin	Arthralgias: jaw, arm; Fatigue, memory problems	3 months	Amoxicillin 4 weeks	95% improved. No relapse
4	Unknown	Back pain	18 months	Unknown	Unknown
5	Spider bite, Rx with erythromycin	Arthralgias, Poor memory; Night sweats	15 months	Ceftriaxone 3 weeks; Amoxicillin 4 weeks	Improved. Relapsed
6	Unknown	Memory loss w/normal CSF dysthesias	6 months	Doxycycline 12 weeks	No improvement.
7	Viral symptoms with rash Rx with 5 day Zithromax	Arthralgia; Fatigue	6 months	Doxycycline 3 weeks	80% improved. Relapsed
8	Unknown	Leg myalgia	12 months	Doxycycline 12 weeks	No clinically significant improvement
9	Unknown	Arthralgias; Olecranon bursitis	12 months	Doxycycline 6 weeks	Marked improvement
10	Remote untreated EM 25 years prior	Memory loss	12 months	Doxycycline 4 weeks	No improvement
11	Viral like illness	Arthralgias; Olecranon bursitis, Edema	7 months	Doxycycline 3 weeks	Complete response. Relapsed
12	Round rash, no treatment	Arthralgias	30 months	Doxycycline 4 weeks	Improvement. Relapsed
13	Unknown	Fatigue, arthralgias, vertigo	18 months	Doxycycline 8 weeks	50% improved. No relapse

Following illness onset, a significant number of all patients had been exposed to glucocorticosteroids, antibiotics not recommended for the treatment of Lyme disease or of less than 10 days duration, or both. There were no significant differences across groups in the proportions exposed to ineffective antibiotics or steroids.

**Figure 3:**
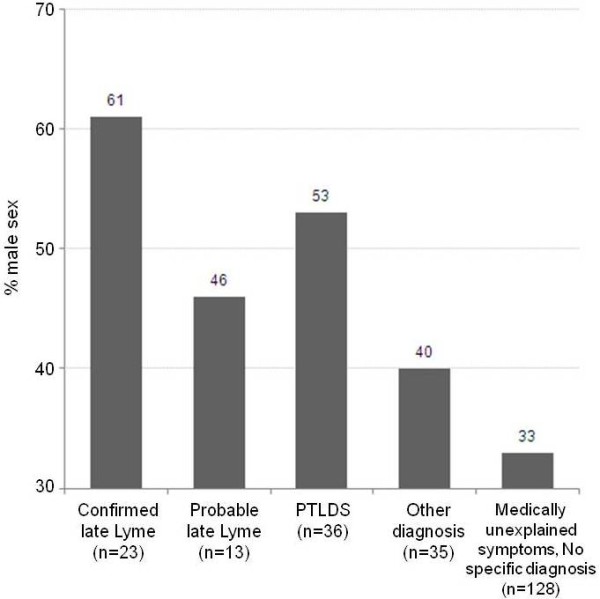
Percent male in each disease group (n = 235).

While male and female patients were approximately equally represented in the probable late Lyme group, the distribution was more variable across groups. Figure 3 shows the percentage male across all five groups, with women slightly under-represented in the first and third groups and over-represented in the second, fourth, and fifth groups. The group distribution was found to be different by sex at trend level of p = 0.06. When compared across the three Lyme-related disease groups (late, probable late, and PTLDS), percent male was not found to be significantly different by group, nor was it significantly different when compared across the two remaining non-Lyme-related groups (other diagnoses and no specific diagnoses). However, when treated as two composite variables, a significantly higher proportion male was found in the Lyme-related group compared to the non-Lyme-related group (p = 0.006). Age at illness onset was also found to be significantly higher in the Lyme-related group compared to the non-Lyme related group (PTLDS p < 0.001).

**Figure 4:**
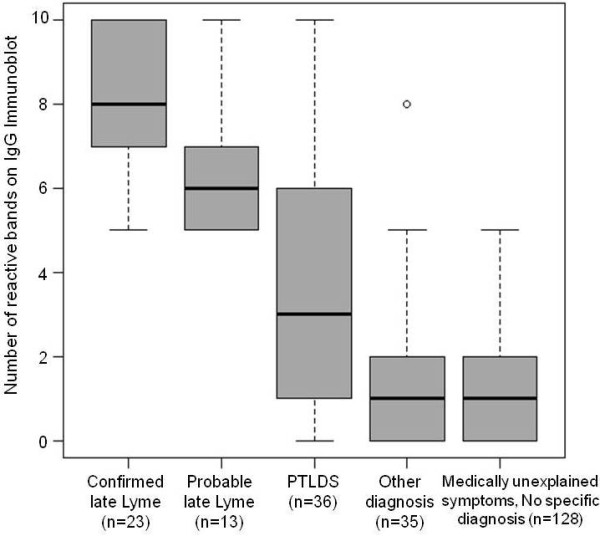
**Box plots of the number of reactive IgG bands on commercially available serologic tests for antibodies to*Borrelia burgdorferi*by disease group (n = 235).**

Figure 4 presents box plots of the number of positive IgG bands present on serologies obtained from patients in each of the five disease groups. By definition, patients with probable late Lyme disease were strongly IgG immunoblot positive by CDC criteria (greater than 5 bands present). PTLDS patients presented with a wide range of number of IgG bands present, whereas those in the final two groups all presented with negative IgG results.

Table 2 shows the specific symptoms and response to therapy of the 13 patients with probable late Lyme disease. Twelve of the 13 patients were treated after the diagnosis of probable late Lyme disease, 8/12 by their referring physician prior to patient evaluation. Of those treated, 75 percent reported what was considered to be clinically significant improvement following antibiotic treatment for probable late Lyme disease. However, 4/9 (44%) of patients who were seen in follow up after treatment had relapse of some or all of their initial pre-treatment symptoms.

## Discussion

This article presents a sample of untreated patients with a history of persistent, subjective symptoms and IgG antibodies to *B. burgdorferi*, a group which has not been well-described. Patients with this presentation make up an unknown proportion of all patients presenting for evaluation of Lyme disease to a general practitioner or medical subspecialist. In our experience, this group accounted for 6% of all patients with symptoms greater than 12 weeks who presented for evaluation of Lyme disease in an endemic region, and 36% of patients with confirmed or probable late Lyme disease.

Patients with this clinical presentation have been considered to be most similar to other subsets of patients with the nonspecific label of “chronic Lyme disease” and thus thought not to have evidence of active, untreated infection with *B. burgdorferi*[12]. As a result, the etiology of their symptoms and any recommendations for treatment remain controversial. However, this group of narrowly defined patients with ongoing symptoms and a positive IgG immuno serology would meet CDC surveillance criteria for ‘probable’ Lyme disease. In the clinical context of months of ongoing symptoms, lack of an alternative diagnosis, lack of prior antibiotic therapy, and a positive IgG immunoblot blot serology, we propose that this group of patients has untreated, probable late Lyme disease.

A comparison of probable late Lyme and confirmed late Lyme patients in our sample show some similarities. Patients in both groups have significant immunoblot reactivity on IgG immunoblot blot analysis, exceeding the highly specific CDC criteria 5 band cut-off for positivity. This pattern strongly supports the exposure of both groups to *B. burgdorferi* and is consistent with a diagnosis of late, untreated infection. The finding of a positive serology by itself is not diagnostic of active, untreated infection in probable late Lyme disease, as it is also present in the convalescent phase of resolved Lyme disease in the estimated 40% of individuals who are never treated during the acute phase and never develop late manifestations of Lyme disease. However in the context of otherwise unexplained symptoms it is a reasonable hypothesis that the patients’ symptoms are a result of previously untreated infection with *B. burgdorferi*. By definition, probable late Lyme disease is characterized by patient-reported symptoms rather than objective physical exam signs of disease, distinguishing this group from those with untreated late Lyme arthritis or neurologic disease. However, the type and chronicity of symptoms in the probable group are very similar to a subset of those patients described in an early observational series of untreated Lyme disease in which 18% of patients developed periarticular or musculoskeletal pain for as long as 6 years, but never developed objective joint abnormalities [11]. In the same study, other patient-reported symptoms including fatigue (41%), headache (16%), myalgias (9%), and abdominal pain (9%) were also found in addition to objective signs of Lyme arthritis.

These early descriptions suggest that the distinction between subjective, patient-reported symptoms and objective signs of late Lyme disease may not be absolute. For example, patient-reported symptoms of musculoskeletal pain may precede the onset of objective synovitis. In addition, the physical finding of joint swelling may be intermittent, so that on any given physician evaluation the only evidence of late Lyme disease may be the patient’s report of symptoms [14].

Further, in probable late Lyme disease, the lack of physician-documented signs of early disease, including EM, is not unexpected and does not necessarily negate the possibility for development of late infection. It is known that during the acute phase of infection, EM may not occur, may not be seen, or may be misdiagnosed, allowing patients to progress to later stages of disease [15,16]. Series of patients with late Lyme arthritis report a history of EM in 23% of patients and “flu-like illness alone” in 16% of patients that preceded their diagnosis of late Lyme arthritis [17]. Interestingly, approximately one third of patients in our series with probable late Lyme disease reported a history of a rash at illness onset that was never physician-documented or treated.

Until the causal relationship of symptoms to infection in probable late Lyme disease can be proven pathologically, the practicing physician must weigh the relative risk and benefit of antibiotic treatment in this group of patients. The finding that 8/12 patients with probable late Lyme eventually received antibiotic treatments for Lyme disease prior to their subsequent referral for evaluation demonstrates that current community practice is often to treat these patients. The treatment approach to this group of patients has not been clearly outlined in the medical literature, with one recent review suggesting that any benefit from treatment would be unlikely [12]. The result that 75% of probable late Lyme patients reported clinically significant improvement with appropriate Lyme disease treatment is consistent with treatment outcomes in confirmed late Lyme arthritis, in which 90% of patients respond to one or more courses of antibiotic therapy. 10% of patients with definite late Lyme disease do not completely respond to antibiotics and are described as having antibiotic-refractory late Lyme arthritis. The pathophysiology of this syndrome is not well understood, although proposed to be an autoimmune based process [17], the role of ongoing bacterial infection in the process remains under investigation [18,19]. The long term outcomes of patients with persistent symptoms after antibiotic treatment of probable late Lyme disease are unclear. In our small case series 4/9 (44%) of patients had relapse of symptoms after their initial antibiotic therapy suggesting that a subset of patients with probable late Lyme disease will go on to develop PTLDS. Future studies are needed to assess effective treatment modalities in a controlled fashion.

Our sample of probable late Lyme disease also showed some differences and some similarities when compared to patients with PTLDS. Patients with probable late Lyme disease had similar rates of patient reported symptoms of pain, fatigue, and cognitive complaints as those patients with PTLDS. In contrast to those with PTLDS, our group of probable late Lyme patients had not been previously diagnosed with Lyme disease, nor had they been previously treated with antibiotics for Lyme disease. The higher rates of IgG seroreactivity in patients with probable late Lyme disease is also in contrast to patients with PTLDS, who all have a history of physician-documented early Lyme disease and variable serologic reactivity. The lower rate of seroreactivity among those with PTLDS is likely explained by exposure to early, effective antibiotics during acute infection, which is known to potentially blunt serologic response to infection with *B. burgdorferi *[20]. It is also in contrast to patients with other, non-Lyme diagnoses and those with medically unexplained symptoms, all of whom lacked IgG immunoblot seropositivity at a level to meet the CDC surveillance criteria for Lyme disease. Until a gold standard with high sensitivity for exposure to *B. burgdorferi* becomes available the percentage of patients with medically unexplained symptoms that are due to exposure to *B. burgdorferi* infection will remain unknown. Because of the limitations of serology in documenting prior exposure to *B. burgdorferi* with early antibiotic treatment Lyme disease, the case definition for PTLDS is clinically based. In an attempt to increase specificity the definition may lose sensitivity by excluding patients with onset of symptoms greater than 6 months after the antibiotic treatment or by excluding those with common pre-existing conditions such as mild depression. As many patients with no specific diagnosis and medically unexplained symptoms had poorly documented past medical histories, we cannot rule out that some may have had unrecognized or undocumented early Lyme disease that was treated, resulting in unrecognized PTLDS.

The observed demographic differences found across disease groups in our sample also warrant further research. The younger, female predominance in the medically unexplained group may reflect the inclusion of patients with syndromes such as fibromyalgia and chronic fatigue syndrome, which are known to have a female predominance. However, the possibility remains unexplored that certain group inclusion or exclusion criteria, or other factors such as patterns of interaction with the health care system, may be associated with specific demographic characteristics.

Previous recommendations have stated that the pre-test probability for Lyme disease in patients without a history of objective manifestations is too low to justify testing and treatment [2]. Recommendations for Lyme disease testing in patients without objective physical findings have been based on assumptions that the incidence of disease in this population is low and not significantly higher than in the general population of a low-moderately endemic region (0.1–0.01% pre-test probability estimates). Our results suggest that the pre-test probability may be significantly higher than these estimates in patients with symptoms being evaluated in a Lyme endemic area; closer to the 6% assumption which has been used for patients with fibromyalgia-like symptoms from a very high incidence region. This assumption of higher disease prevalence leads to a post-test probability of approximately 25%, more than twice what has been reported in previous analyses [2]. Among those with probable late Lyme disease, a patient-reported history of rash suggests that the pre-test probability of Lyme disease (and thus the predictive value of a positive serologic result in certain selected patients with very significant histories) may be even higher. We suggest that in patients from Lyme endemic regions, the possible diagnosis of probable late Lyme disease is reasonable to consider in the setting of an unexplained illness and a history highly suggestive of Lyme disease exposure. These patients may benefit from testing for IgG antibodies to confirm exposure to *B. burgdorferi* and to suggest the possibility of late untreated infection.

There are several important limitations to this study and future research is warranted. First, the retrospective nature of the data relied largely on patient self-report as well as on serologic results from several commercial laboratories and past medical records from several physician offices. Because of this, results of testing for other tick-borne infections such as babesiosis, anaplasmosis, ehrlichiosis, bartonellosis, and rickettsiosis. were generally not available and were not included in this report. The insensitivity of serology for the early diagnosis of Lyme disease and for documenting remote exposure to *B. burgdorferi* infection may have led to unintended misclassification of an unknown number of cases currently defined as medically unexplained and led to an underestimation of the true numbers of cases of Lyme disease or PTLDS. Extrapolation of our findings in patients will probable Late Lyme disease to the much larger number of patients with “syndrome” diagnosis such as fibromyalgia, chronic fatigue syndrome, and medically unexplained symptoms with be the focus for future investigations when better biomarkers for Lyme disease and *B*. *burgdorferi* exposure becomes available.

In addition, the possibility of sex based differences in performance of serologic tests for Lyme disease may further complicate the ability to establish exposure to *B. burgdorferi* and to make the accurate diagnosis of Lyme disease. The opportunity exists for recall or other biases, particularly among patients eager to label previously unexplained symptoms. While the retrospective nature of the data is not ideal, we would argue that the opportunity for prospective studies capturing this subset of patients is challenging. Finally, because of the small sample size in our case series, additional studies with larger sample sizes are needed to see if our findings are replicable.

## Conclusions

In conclusion, untreated patients with persistent, unexplained symptoms and serologic evidence of prior exposure to *B. burgdorferi* represent an interesting subset of patients presenting for evaluation of Lyme disease to a community based Lyme disease referral practice. These patients with probable late Lyme disease have a combination of patient reported symptoms such as fatigue, widespread pain, and cognitive complaints but lack the classic objective findings of joint synovitis or large fiber neuropathy that defines definite late Lyme disease. Some of these patients may have a history suspicious for previously unrecognized early Lyme disease with EM, which highlights the importance of careful physician histories in endemic regions. The predictive value of a positive serologic result in this subset of patients may not be as low as previously thought. Physicians should consider the recent inclusion of probable Lyme disease in the CDC Lyme disease surveillance criteria when evaluating patients, especially those with a history consistent with misdiagnosed or inappropriately treated early Lyme disease. Further studies are needed to more precisely define the clinical spectrum, ideal treatment, and long-term outcomes of patients with probable late Lyme disease.

### Consent

Written informed consent was obtained from the patient shown in Figure 2 for publication of this manuscript and any accompanying images. A copy of the written consent is available for review by the Series Editor of this journal.

## Competing interests

The authors declare that they have no competing interests.

## Authors’ contributions

JA conceived of the study, participated in its design and coordination and helped draft the manuscript. AS assisted with the data analysis and helped draft the manuscript. AR participated in the study design, performed the statistical analysis and helped draft the manuscript. All authors read and approved the final manuscript.

## Pre-publication history

The pre-publication history for this paper can be accessed here:

http://www.biomedcentral.com/1471-2334/12/173/prepub
